# Trust but Verify: Lessons Learned for the Application of AI to Case-Based Clinical Decision-Making From Postmarketing Drug Safety Assessment at the US Food and Drug Administration

**DOI:** 10.2196/50274

**Published:** 2024-06-06

**Authors:** Robert Ball, Andrew H Talal, Oanh Dang, Monica Muñoz, Marianthi Markatou

**Affiliations:** 1 Office of Surveillance and Epidemiology Center for Drug Evaluation and Research US Food and Drug Administration Silver Spring, MD United States; 2 Jacobs School of Medicine and Biomedical Sciences Buffalo, NY United States; 3 School of Public Health and Health Professions University at Buffalo Buffalo, NY United States

**Keywords:** drug safety, artificial intelligence, machine learning, natural language processing, causal inference, case-based reasoning, clinical decision support

## Abstract

Adverse drug reactions are a common cause of morbidity in health care. The US Food and Drug Administration (FDA) evaluates individual case safety reports of adverse events (AEs) after submission to the FDA Adverse Event Reporting System as part of its surveillance activities. Over the past decade, the FDA has explored the application of artificial intelligence (AI) to evaluate these reports to improve the efficiency and scientific rigor of the process. However, a gap remains between AI algorithm development and deployment. This viewpoint aims to describe the lessons learned from our experience and research needed to address both general issues in case-based reasoning using AI and specific needs for individual case safety report assessment. Beginning with the recognition that the trustworthiness of the AI algorithm is the main determinant of its acceptance by human experts, we apply the Diffusion of Innovations theory to help explain why certain algorithms for evaluating AEs at the FDA were accepted by safety reviewers and others were not. This analysis reveals that the process by which clinicians decide from case reports whether a drug is likely to cause an AE is not well defined beyond general principles. This makes the development of high performing, transparent, and explainable AI algorithms challenging, leading to a lack of trust by the safety reviewers. Even accounting for the introduction of large language models, the pharmacovigilance community needs an improved understanding of causal inference and of the cognitive framework for determining the causal relationship between a drug and an AE. We describe specific future research directions that underpin facilitating implementation and trust in AI for drug safety applications, including improved methods for measuring and controlling of algorithmic uncertainty, computational reproducibility, and clear articulation of a cognitive framework for causal inference in case-based reasoning.

## Introduction

A very common task accomplished by medical professionals many times a day is reasoning about an individual case to make medical decisions. Supporting case-based reasoning with automation for most clinical situations remains a challenge despite advances in artificial intelligence (AI) [1]. To be most successful, predictive “AI” (eg, machine learning [ML]) requires large amounts of annotated data and will, therefore, perform best in large-scale clinical situations where such annotation is possible (eg, image interpretation). However, most of clinical medicine (1) consists of situations that, while common, are very complex, making the identification of all the necessary predictive features difficult; (2) is small-scale, where there is incomplete understanding about what the important features are; and (3) can involve genuine uncertainty in the application of consensus guidelines to an individual patient. Developing strategies for how best to apply AI to these situations is essential to fulfill the potential of AI to assist with patient care.

Case-based reasoning about a drug’s risks is a key component of the assessment of postmarketing individual case safety reports (ICSRs) at the Food and Drug Administration (FDA) [2]. An ICSR contains a description of an adverse drug experience related to an individual patient prepared in a standard format for submission to the FDA and other regulators. Information about the risks of a drug tend to increase after the drug is approved and used by large numbers of patients. While some of this information comes from postmarketing trials and observational studies required by the FDA and observational studies conducted in the FDA’s Sentinel System [3], a critical source of information is case reports of adverse events (AEs) from individual patients and their providers [4-7]. The process of assessing these ICSRs is akin to a clinician’s task of considering multiple possibilities to assign a diagnosis and includes the identification of key features of the drug, the clinical events, the temporal relationship between drug exposure and the clinical events, and demographic features of the patients. These factors must be interpreted against a background of past medical history, other medications and exposures, and the natural history of the disease being treated. The goal of this process is a decision as to whether it is likely the drug in question caused the observed clinical events.

To make this process more efficient and improve its scientific rigor, the FDA embarked on a program to develop automation support for ICSR assessment [8]. In this viewpoint, our aim is to describe the lessons learned from this experience and additional research needed to address both general issues in case-based reasoning using AI and specific needs for ICSR assessment. We first describe the FDA’s experience applying natural language processing (NLP) and ML to ICSR assessment and the recognition that, for automation support to be successful, it must have the full trust of those it is supporting. Then, we apply the Diffusion of Innovations theory to the FDA’s experience to illuminate the sociotechnical reasons for FDA safety reviewers’ acceptance of one AI algorithm (ie, the process of deduplication) but not another (ie, assignment of causal relationships) [9]. This analysis leads to the recognition of the importance of a formal inferential framework for ICSR assessment. We conclude with a discussion of the need for a deeper understanding and potential reframing of the cognitive framework used for causal inference and research priorities for AI to be fully applied to case-based reasoning and clinical drug safety assessment.

### FDA’s Experience in Applying NLP and ML to ICSR Assessment

Analyzing ICSR data is challenging because of the limitations of these data, including the underreporting of AEs and the lack of accurate data on drug use preventing the calculation of accurate AE occurrence rates; the lack of controls; as well as data limitations within the reports themselves, including missing, imprecise, or occasionally inaccurate clinical information. These and other drawbacks limit the usability of current methods to draw a causal link between a reported drug and the AE based on statistical properties of their occurrence in an AE database alone.

**Figure 1:**
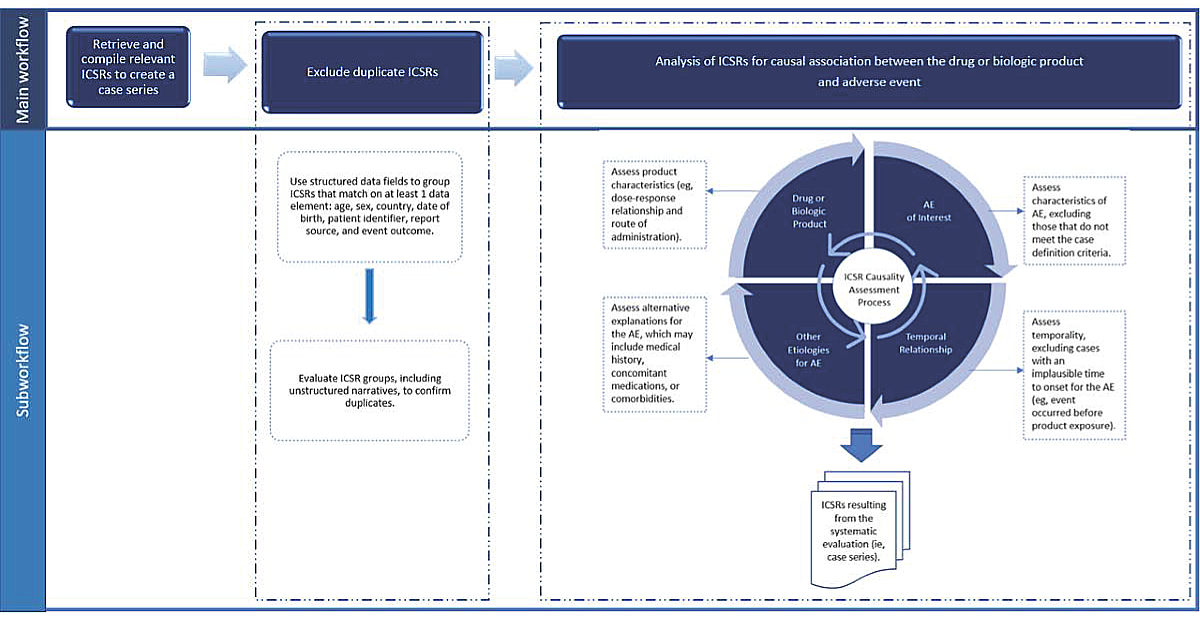
Individual case safety report (ICSR) evaluation workflow. This figure illustrates the ICSR evaluation workflow from the retrieval of ICSRs to the creation of a case series that has been deduplicated and assessed for causality. The assessment for causality uses case-based reasoning and considers information from the ICSR about the drug or biologic product, adverse event, temporal relationship, and other etiologies for the adverse event. The output from the ICSR causality assessment process is a case series of ICSRs that have been evaluated for causal association.

The assessment of ICSRs for possible causality still relies primarily on expert judgment and global introspection. Figure 1 provides an overview of the ICSR evaluation workflow (Figure 1 is adapted from the study by Ball and Dal Pan [8]. Case definitions are a set of prespecified criteria for determining whether a patient should be identified as having a particular disease, injury, or other health condition (ie, AE) [2]). Current practice for drawing inferences from these data involves expert review of case series and comparison with external sources of information (eg, product labeling describing known AEs and pharmacological mechanisms) supplemented with summary statistics and disproportionality scores [2,10,11]. Important clinical information (eg, temporality, concomitant medications, comorbidities or past medical history, or alternative explanations for the AE) is typically found in the ICSR narrative, which is generally believed to be the key to making an accurate assessment, provided that the narrative contains the relevant information. This means that the application of AI techniques must focus on methods to extract and organize meaningful information from these narratives.

The initial development of this approach at the FDA was conducted using reports to the FDA and Centers for Disease Control and Prevention’s Vaccine Adverse Event Reporting System focusing on automating the identification of 2 rare, but important, vaccine AEs, anaphylaxis and Guillain-Barre syndrome, as test cases [12-14]. The first step was to develop an NLP approach to select and extract clinical manifestations and demographic characteristics of the patients for the clinical condition of interest. Subsequent work involved further development of the text mining system to extract drug or biologic product exposure, temporality, and alternative explanations for the AE [15-17]. Finding duplicate reports of the same case (ie, if the same AE instance for the same individual is reported by multiple reporters) is an important practical step in the assessment of ICSRs. While algorithms based on structured fields in the ICSRs have been available, an important advance occurred with the incorporation of clinical text extracted using NLP from the case narratives into the algorithm [18-20]. In these examples, the features were extracted from narratives primarily using rule-based approaches.

A complementary approach to the aforementioned one is to take a holistic view of the causality assessment process and allow the machine to identify the key features of a report. Using relatively small subsets of ICSRs from the FDA Adverse Event Reporting System classified on a 5-point scale as to the likelihood that the drug caused the AE, supervised ML was used to predict these classifications [21,22]. Another approach used logistic regression to predict the likelihood that an ICSR contained useful information by its inclusion in an FDA case series review supporting a recommendation to modify product labeling [23]. These efforts demonstrate the feasibility of developing models to predict which ICSRs most likely contain information for a human safety reviewer’s triage assessment of the report. However, the algorithm’s performance is not sufficient to allow for use without human expert review of the predictions.

The relative success of these algorithms led to their incorporation into decision support tools [24,25]. The experience using these tools in operational pilots is described in the following section.

### The Importance of Trust in Building AI Support for ICSR Assessment

User trust in AI can be furthered through close collaboration among end users and AI developers in an iterative process [26]. Continuous communication between these stakeholders is needed to understand pharmacovigilance problems and uncover challenges as experienced by safety reviewers, the selection of use cases for AI, the development of required functionalities to support the use case, and appropriate insertion of AI in the pharmacovigilance workflow. Safety reviewers need to have confidence that AI developers sufficiently understand the complexity of the pharmacovigilance problem to propose whether and which AI methods can be optimally applied to address the specific problem. The ICSR evaluation workflow is complicated by many and varied tasks; the consideration of a multitude of features that require interpretation for decision-making; and the application of clinical domain expertise in the context of the use case, such as duplicate detection, clinical classification, or causality assessment. A shared understanding of the intricacies involved in ICSR evaluation supports safety reviewers and AI developers working together to define appropriate use cases and the approach for integration in the pharmacovigilance workflow.

Humans have rich prior knowledge. The engagement of the safety reviewer with the computer algorithm can facilitate addressing the issue of incomplete features and data sparseness, especially when there is a lack of training data, as is the situation with many ICSR assessments and clinical decision-making. The concept of “human-in-the-loop” has been proposed to tackle these challenges, by incorporating human knowledge into the process and addressing the AI algorithm’s limitations.

### Acceptability of NLP and ML Algorithms by Safety Reviewers and the Diffusion of Innovations Theory

Despite what would appear to be adequate performance to provide an aid for improving ICSR review, the software tools initially were met with limited acceptance by safety reviewers, although each development cycle included their active involvement in the collection of requirements and testing of user interfaces.

Discussion with safety reviewers about AI for causality assessment was met with skepticism because their perception is that this problem is challenging for humans, let alone machines. In the previously mentioned FDA study [21] classifying reports on a 5-point scale as to the likelihood that the drug caused the AE, at least 2 of the 3 adjudicators tended to agree as to a report’s classification. When the reports were regrouped to create a binary classification (ie, Certain, Probable, or Possible vs Unlikely or Unassessable), all 3 adjudicators agreed on 61% of the reports [21].

In contrast to the causality assessment use case, duplicate ICSR detection was perceived by the safety reviewers as a high priority and a potentially solvable problem for AI. Duplicate ICSRs contribute to inefficiencies by increasing workload because they are generally identified via manual processes but can be relatively easily verified if presented by an algorithm. Trust-building efforts, aligning with the safety reviewers’ workflow and communication, were applied to explicitly map clinical features used in the safety reviewers’ current manual deduplication processes to that of the AI-based algorithm. In addition, the NLP-extracted common clinical features and their effects on the duplicates identified by the algorithm were further visually presented to enhance end users’ understanding of the features that were used by the algorithm to identify duplicates.

Feedback from safety reviewers [27] suggested several reasons for their initial limited acceptance for causality assessment when performed using AI. First, the AI approach is outside of their domain of expertise and clinical training. Furthermore, the approach did not offer sufficient explanation or flexibility to accommodate information external to the ICSR (eg, reviewer’s knowledge). The software tools were not fully aligned with the reviewer’s current business practices and workflows. Because of the lack of trust in the software tools, any efficiency gains, compared to current business practices and workflows, were not perceived as making it easier for the safety reviewers to assess whether the drug caused the outcome described in the report.

### Ethical Considerations

FDA employees were asked to provide feedback about their experience with the deduplication algorithm as part of a project conducted in collaboration with Johns Hopkins University under the FDA Centers of Excellence in Regulatory Science and Innovation program. The Johns Hopkins Medicine Institutional Review Board determined that the project did not constitute human subjects research under the US Department Health and Human Services or FDA regulations. As this was not considered human subjects research, informed consent was not obtained. All participation in surveys was voluntary and was part of routine employment at the FDA; hence, participants did not receive additional compensation. Survey participants provided consent to use their anonymized responses in the manuscript.

### Diffusion of Innovations Theory

Diffusion of Innovations is a well-known theory that has been applied to explain how new ideas and technologies are adopted [9]. According to the theory, several attributes influence the rate of spread of an innovation and can offer insight into the FDA safety reviewers’ acceptance of AI algorithm use (Table 1).

In Table 1, the acceptability of the application of AI algorithms to ICSR deduplication is assessed through the main attributes, including the social system, the communication channels used for dissemination, and the attributes of the innovation. This example illustrates how the AI algorithm for deduplication successfully incorporated the key attributes of an innovation, namely, relative advantage, trialability, and observability. The successful fulfillment of these attributes promoted trust in its use. The key to its acceptance is that the tool transparently implemented an algorithm that closely paralleled existing pharmacovigilance workflows and safety reviewers’ cognitive process for deduplication.

In contrast, 2 attributes of innovation, compatibility and complexity, offer a possible explanation for the lack of acceptance of the causality assessment algorithm. Causality assessment is much more complex than duplicate detection, as illustrated by the larger number of attributes required for consideration and their interactions, listed on the right side of Figure 1. The determination of the likelihood that a drug caused an AE in a report is considered the most challenging aspect of a safety reviewer’s work. Not only are the drug exposure and the AE outcomes important considerations, but alternative explanations of the phenomenon and temporality must also be considered. The social environment in which safety reviewers work encourages complete and transparent characterization of all evaluated ICSRs following a deterministic logic. Using probabilistic predictions in such an environment requires either perfectly performing algorithms or a validation process that limits the benefits of the algorithms. While the same issue arises in duplicate detection, the validation process is simpler, and the consequences of incorrect duplicate classification are lower than those for causality assessment. The existing technical environment in which safety reviewers work offers no other applications of computing to address a problem of similar complexity. In fact, many simpler problems remain unautomated, for example, automatically flagging an AE as being present in the drug’s package insert, leading to the perception that it is unlikely that a machine could successfully help solve the more complex problems.

Furthermore, 2 other “diffusion of innovations” attributes, the ability to readily observe an intervention’s functionality (ie, observability) and to readily experiment with it (ie, trialability), are also likely important. The relative difficulty of observing the process by which an ML algorithm assigns a causality assessment may complicate safety reviewer acceptance. While the general parameters of the approach to causal inference in ICSR evaluation have been described [2,10,11], no complete articulation of all the data elements and their interrelationships in the form of an algorithm has been accomplished. This lack of a basis for verification of an algorithm’s validity suggests that developing a more complete understanding of how inferences are made in case-based reasoning is an important next step. To increase acceptance of ML approaches to causality assessment, more work is also needed on how to best fit causality assessment algorithms into safety reviewer workflows to allow them to try out the results of the algorithms without total commitment.

**Table 1:** Diffusion of Innovations theory applied to the Food and Drug Administration’s AI^a^ algorithms.

Attribute (definition)	Deduplication		Causality assessment	
	Relevance	Evidence	Relevance	Evidence
Social system (interconnected units working collaboratively toward common goal)	Duplicate ICSRs^b^ are viewed as a common problem by safety reviewers during their case series evaluation.	Participants viewed themselves as a community of safety reviewers in which their key role involves evaluating ICSRs for drug-related safety issues. During this process, they experienced the challenges of efficiently identifying duplicate ICSRs for exclusion from the case series (Figure 1).	Assessing causality during ICSR evaluation involves evaluating the likelihood and strength of the relationship between a drug and an adverse event at a report level. Causality assessment is a critical component of safety signal management.	Consistent with established practices for safety surveillance, safety reviewers assessed ICSRs before assembling a case series that includes cases assessed as causally associated. Reviewers documented a summary of the considerations or rationale for inclusion of the ICSRs in the case series [2].
Communication channel (method of information spread; users’ ability to perceive usefulness)	Safety reviewers were involved in iterative requirements gathering processes. They provided continuous input during the testing and evaluation of the deduplication tool.	Verbal and written communication provided an explanatory description of the deduplication algorithm tool and where it could fit into the safety reviewers’ workflow. Multiple rounds of feedback were collected during the testing and evaluation of the deduplication algorithm tool.	As part of a series of research and development efforts to implement a tool to support case series evaluation that include causality assessment, interactive meetings with multiple groups of safety reviewers were held to understand current practices and workflow for conducting causality assessments.	Discussions with safety reviewers revealed concerns about the effectiveness and utility of a one-size-fits all algorithm that classifies ICSRs by level of causality. Safety reviewers viewed causality assessments as a complex task for both humans and AI. As a result, they did not prioritize causality assessment for incorporation in a tool to support case series evaluation.
Attributes of innovation: relative advantage (perception of benefit or improvement over existing technology)	Safety reviewers compared the usefulness and efficiency gained from the automated deduplication algorithm output against that of the current baseline, which is to manually use spreadsheets to find duplicate ICSRs.	“I think the deduplication method could be used instead of the current process, as the algorithm did find some cases that would have been missed. However, the cases still need to be screened to determine if the cases are duplicates or not.” “Really a big help to save time.” “It is helpful that the algorithm grouped likely matches together; this saved some time.”	The existing approach for conducting causality assessments is a complicated manual process that involves many steps. In particular, it requires clinical and pharmacovigilance expertise.	Safety reviewers did not view the automation of drug causality assessment as a relative advantage over the current manual process. Rather, there was skepticism around whether and how well AI could emulate human experts’ thinking in terms of applying clinical knowledge and judgment to accurately conduct a causality assessment of ICSRs.
Attributes of innovation: compatibility (consistent with existing technical and social environment)	The deduplication tool is consistent with and supports the current technical, business process, and safety reviewers’ workflows.	“Usability was very straightforward, easy to run.” “Very quick turnaround time (minutes) allowed me to start working on it right away.” “I will continue to use the algorithm.” “This tool doesn’t replace my deduplication process but is a helpful addition.”	Causality assessments to detect and evaluate the relationship between a drug or biologic and adverse event of interest are inherently part of the safety reviewer’s workflow and processes.	The use of AI for causality assessments is viewed by safety reviewers as a complicated task that is likely not yet solvable by current technology. Particularly, there are nuances and factors that need to be considered in various use cases to which causality assessments are applied. A question remains whether the AI output for causality assessment could be generalized to any drug and adverse event of interest. In addition, it is not clear how the AI output could be usefully incorporated in the current workflow to support safety reviewers.
Attributes of innovation: complexity (perception of the difficulty of implementation, use, or understanding)	During the manual process of detecting duplicate ICSRs, safety reviewers conducted a stepwise comparison of data points from structured fields, followed by those from the narratives between potential duplicate pairs to find actual duplicate reports during case series evaluation.	The deduplication tool considers multiple relevant features from ICSR structured fields and narratives, many of which overlap with those used in the safety reviewers’ best practices deduplication processes. Most safety reviewers stated that they were very likely or likely to use the deduplication algorithm for their reviews and had medium to high confidence in the deduplication algorithm tool’s output.	The processes involved in causality assessment require more advanced logical reasoning and considerations of interrelationships among various data in the structured and unstructured information from ICSRs. Furthermore, clinical and pharmacovigilance knowledge and expertise is applied during causality assessment (ie, external information that may not be represented within an ICSR).	Safety reviewers participated in successive research focused on applying AI for causality assessments. To train and test the ML^c^ classification algorithm for causality, safety reviewers created a reference data set of annotated ICSRs based on the likelihood that the drug caused the adverse event. The ambiguity and complexity of annotating levels of causality was illustrated by the substantially lower interannotator agreement among 3 adjudicators compared to that for 2 adjudicators. The performance characteristics of the ML algorithms for causality were perceived as not being adequate. Moreover, safety reviewers do not have domain expertise in ML, which could affect the perceptions and understanding of the strengths and limitations of technology. When the ML algorithm used certain features not used by human safety reviewers, it was perceived as a limitation. In addition, the ML causality classification algorithm did not use external data sources, such as clinical knowledge of the medical history or concomitant medications, that safety reviewers routinely apply.
Attributes of innovation: trialability (ability to try without total commitment and with minimal investment)	A 6-month study allowed all safety reviewers to test and evaluate the usefulness of the deduplication algorithm within their current workflow and provide additional feedback.	“It’s a good backup and second check to my own deduplication.” “It’s a good second check.” “Although the algorithm doesn’t replace my own deduplication, I find it helpful in combination with my process.”	Safety reviewers were offered a plausible workflow option that would incorporate the output of the ML for causality assessment in their workflow process. The option was to use the ML algorithm’s output to prioritize the review of ICSRs that were classified with the highest likelihood of a causal association, followed by those with lower likelihood of causality.	Despite the development of the ML algorithm for ICSR classification of causality, the proposed plausible workflow would still need considerable time and human resource investment. First, from the safety reviewers’ perspective, the ML algorithm did not preclude the need to conduct causality assessments, the most resource intensive step, for all the ICSRs within a case series. Second, because ICSR prioritization is a not part of the current workflow, a new workflow that effectively integrates prioritization would need to be developed.
Attributes of innovation: observability (visible benefits to potential adopters)	Safety reviewers were able to experience the application of the deduplication algorithm tool to each of their specific case series of interest within the existing workflow. The benefits of the tool were apparent while using the tool.	“Although I don’t think it saved any time in this data set, the algorithm identified duplicates I would have missed.” “...good for screening large numbers of reports.” “I still believe there is beneficial utility to the tool, possibly with searches producing high caseloads.”	Safety reviewers were presented with the option to use only the final output from the AI algorithm for causality assessment that automates the classification of ICSRs as assessable or not assessable. Assessable ICSRs contain sufficient information for a safety reviewer to be able to conduct a causality assessment, whereas unassessable reports have insufficient information.	Safety reviewers neither completely trusted nor valued the benefit of solely using only the ML classification output of assessable or not assessable in their current workflow. Concerns were raised about the risk of missing an important ICSR because of misclassification by the ML algorithm. Rather than use the ML algorithm’s final classification output, reviewers requested to view and understand components of the algorithm. The potential benefits and how to optimally incorporate this AI output in their workflow remain unclear and thus not observable.

^a^AI: artificial intelligence.

^b^ICSR: individual case safety report.

^c^ML: machine learning.

### The Importance of a Formal Inferential Framework for Building Trust in AI Support for ICSR and Clinical Drug Safety Assessment

What are the key components of a plausible inferential framework for building trust in AI systems for drug safety causality assessment? At the heart of this framework is measuring and managing uncertainty. Additional components include understanding and mitigating the impact of data biases as well as understanding how ML algorithms for drug safety operate, their strengths and limitations, and how they can be applied and tuned for a given task. Furthermore, computational reproducibility is a key component for valid inference. Thus, in our context, the term signifies well-described and standardized workflows, computing environments, and the ability to obtain the same results if the same data and algorithm are used by 2 users following the same workflows. In the following paragraphs, we discuss these aspects in detail.

Traditionally, inference from clinical data has been based on a hierarchy of evidence, with randomized, blinded clinical trials considered the gold standard, while individual case reports, such as those discussed in this paper, are considered to have the least evidentiary value. More recently, there has been a movement away from this “hierarchy” toward a recognition that valid causal inferences can be made from a synthesis of different types of data, enabled by advanced computational techniques including AI [28,29]. Traditionally in pharmacovigilance, certain AEs, such as anaphylaxis, have been attributed to drug exposure after only a few case reports if the following two conditions are met: (1) the time between the drug exposure and the onset of the condition is relatively short and consistent with the known mechanism of action and (2) there are no other obvious causal factors. For anaphylaxis, most reactions occur within minutes to hours after exposure [30]. Progressive multifocal leukoencephalopathy (PML) was attributed to the treatment of patients with multiple sclerosis with natalizumab after only a few cases were observed based on the rareness of PML, the plausibility of natalizumab causing immunosuppression, and the presence of the infectious agent that causes PML [31]. What matters more than the traditional hierarchy is the proper application of the “rules of inference” to data that are “fit-for-purpose.” In the context of drug safety, this means that better understanding of both elements is necessary to make advancements in creating a computable drug safety cognitive framework.

By a drug safety cognitive framework, we mean the rules of inference applied to fit-for-purpose data in ICSRs by safety reviewers to assess whether there is likely to be a causal relationship between an exposure and an AE. As outlined in Figure 1, the data categories included in the framework include the following:

- The drug exposure
- The timing between the exposure and the onset of the AE of interest
- Concomitant exposures, including other drugs and the timing between their exposure and the onset of the AE of interest
- The natural history of the disease and its relationship with the AE of interest
- Prior medical history including other conditions and their natural history and relationship with the AE of interest
- If available, information about dechallenge or rechallenge, that is, if the AE stops when the drug is discontinued and reoccurs when the drug is restarted, can support a conclusion that the likelihood of a causal relationship is increased

Factors external to the information contained in an ICSR include knowledge of the mechanism of action of the exposures and their relationship with the AE of interest, including whether the timing of exposure and onset of the AE of interest is mechanistically feasible, and knowledge of whether the AE is known to be caused by the exposures.

“Fit-for-purpose” data are data suitable to be used for pharmacovigilance and are characterized by the following properties:

- They are fit-for-purpose from a quality standpoint. Are the data statistically sound to be used for the purpose of identifying AEs?
- They are fit-for-purpose from a timing perspective. Are the data current enough to form the basis for the safety question of interest?
- They are fit-for purpose for regulatory action. Whatever is discovered from the data needs to be understood to take action.
- They are fit-for-purpose for representing the population affected by the safety issue. For example, do the data pertain to certain sex, race, age, ethnicity, or other important categorization of the population affected?

The key to understanding the relationships between “rules of inference” and “fit-for-purpose” data, as it relates to the use of AI, is how different approaches manage uncertainty. The pharmacovigilance setting is that of learning from data and, as such, is inseparably connected to uncertainty. Decision-making involves uncertainty. Some of the uncertainty concerns facts. For example, how long does it take to develop anaphylaxis after exposure to a drug that might initiate this event? Taking full advantage of the scientific research dictates knowing its associated uncertainty. Both extremes, that is, too much confidence or too little confidence, are problematic. The first extreme raises the possibility of facing unexpected problems, such as missing important AEs potentially resulting in increased morbidity and mortality. The second extreme raises the possibility of missing opportunities while wasting time and resources, such as unnecessarily conducting additional studies to address a safety issue. In the context of pharmacovigilance, substantially consequential decisions need to be taken with reference to the modification of the drug product labeling of certain medications or even removal of medicinal products from the market. Thus, improving the understanding of sources of uncertainty and their implications for the consequences of a decision is a fundamental need when using AI.

At a high level, there are 2 types of uncertainty: aleatoric or statistical uncertainty and epistemic uncertainty. Aleatoric uncertainty expresses the inherent randomness associated with an observed process, while epistemic uncertainty refers to the uncertainty associated with limited knowledge, which can be partially reduced by increasing knowledge. These uncertainties are of different nature, with aleatoric being a stochastic component in the data generation process and epistemic being associated with the state of our knowledge about a phenomenon of interest. An example of aleatoric uncertainty is coin flipping; there is a stochastic component to the data generation process that cannot be reduced by adding any type of information, while epistemic uncertainty is reducible as more information becomes available. Human performance in the evaluation of ICSRs is assumed to have the minimum amount of irreducible epistemic uncertainty, but in many situations, no baseline is available to inform the threshold for minimum AI performance characteristics. Senge et al [32] referred to the distinction between the 2 types of uncertainties and proposed a quantification that is illustrated in the context of medical decision-making. Another recent work [33] discussed these concepts in the context of ML. At this point, it is important to emphasize that other sources of uncertainty may exist, for example, measurement error, outliers, model uncertainty, or incorrect model assumptions [34].

**Figure 2:**
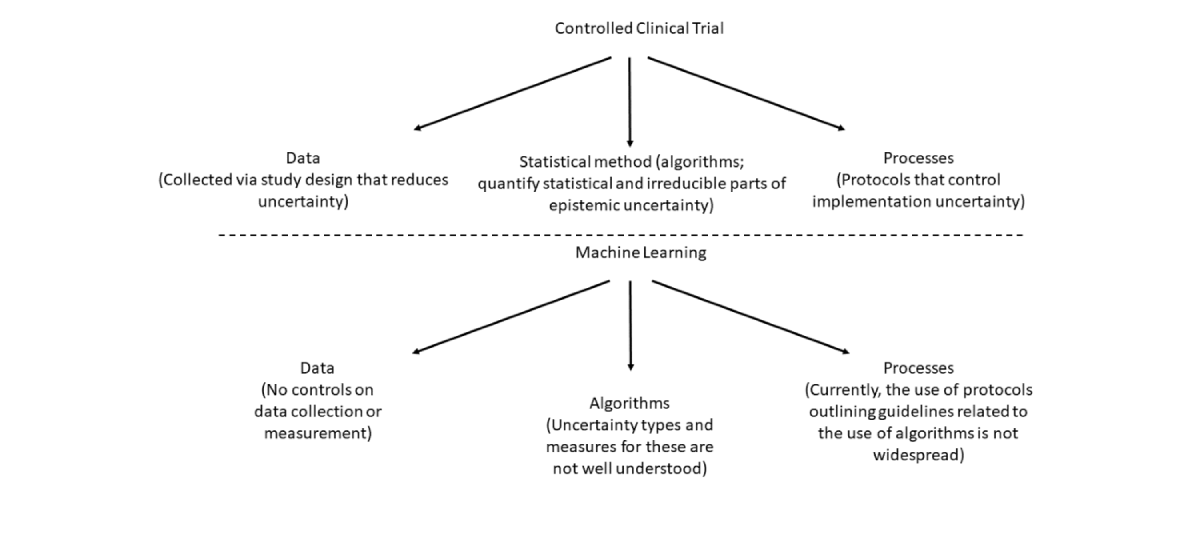
Main aspects of clinical trials and machine learning technologies. Text in parenthesis indicates how uncertainty is controlled. Controlled clinical trials have well-developed protocols that define appropriate processes that aim to reduce statistical uncertainty. Analogous protocols should be developed for machine learning applications.

In the context of clinical trials, Figure 2 indicates the different components associated with the aleatoric versus epistemic uncertainty framework. Each stage of the trial has a clear role in reducing aleatoric uncertainty. For example, the stage labeled as processes is well defined, in that implementation and the analysis of a clinical trial are executed by well-defined, a priori developed protocols. The data collection follows appropriate designs determined before the collection commences. In addition, the entry criteria in the protocol define the population, and randomization seeks to balance predetermined enrollment criteria to ensure the validity of inference. Therefore, the statistical or the irreducible part of the uncertainty is well controlled. Data collected from clinical trials are experimental, are generally carefully generated, and are subjected to many controls.

In contrast, a parallel framework for the use of the ML algorithms in pharmacovigilance does not currently exist. Recognizing that randomized controlled trials are for causal inference at population levels and ML is used for predicting outcomes for individuals, the lessons learned from randomized controlled trials about how well-defined processes can reduce and control statistical uncertainty can facilitate the development of a framework appropriate for ML that, in turn, contributes toward trusting AI systems. We discuss this in the rest of this section. In the context of ML, data are not collected according to any predesigned experiment; these are observational data. The self-controlled case series method uses only cases to study the association between adverse health outcomes and medical products [35,36]. This method can be applied to population-based databases and has been used in vaccine safety studies. However, the use of these designs for the analysis of data that reside in population-based databases has well-known limitations, including the strong assumptions made for the analysis to be possible [35]. ML methods are used to predict outcomes using different data sources. The uncertainty in predictions can then be assessed to provide a measure of trustworthiness of the results.

ML algorithms are not well understood or easily comprehended by their users, as evidenced by the experiences of the safety reviewers discussed earlier. These algorithms depend on data; to identify the circumstances under which they operate implies that the analyst must know and understand the data with which the algorithm works. In addition, many of these algorithms depend upon their hyperparameter setting (ie, a configuration that is external to the model used; they are often set or tuned by the users of the model for the specific problem for which they are applied) to have good performance. The definition of “good performance” itself is subject to discussion (eg, whether to emphasize precision or recall) and depends upon the context of use. Returning to the context of hyperparameters, an algorithm’s performance depends on the setting of these parameters or tuning, an action that requires the definition of a search space [37,38]. This is indeed a serious limitation of these methods. Currently, the FDA is applying ML algorithms to separate out reports that are highly unlikely to contain information that can be used by a safety reviewer to establish causality (ie, likely not useful data) [22].

A proper representation of algorithmic uncertainty is, therefore, an important prerequisite for the acceptance of AI methods and ML algorithms in pharmacovigilance. Quantifying algorithmic uncertainty is a current active research area, with authors attempting to propose measures that reflect aleatoric and epistemic uncertainty [32]. The field of statistical sciences has contributed to the quantification of aleatoric uncertainty; examples include methods that measure the variance of resampling estimates (ie, cross-validation or bootstrap) of the generalization error of computer algorithms [39-42]. Informally speaking, the generalization error is the error an ML algorithm makes on cases that it has not seen previously and indicates the algorithm’s predictive ability. Relatively recent work proposes the construction of CIs for the generalization error [43-45].

An important consideration is the training of the algorithm and the FDA safety reviewers, that is, the individuals who will be using the algorithm. The training of ML algorithms to identify cases that represent AEs requires large amounts of data, which may be a limitation for rare or extremely rare AEs. In the case of training safety reviewers to use ML or rule-based algorithms, trust in the algorithm can be developed by educating end users, such as safety reviewers, about the algorithm. The training should include the features used, how they operate, what their strengths and limitations are, and how the algorithms can be applied to relevant tasks. The more comfortable the reviewers are with the algorithms, the more likely they will be to actually use them.

Algorithms are models and, as such, are imperfect. Box [46] stated that “All models are wrong, some are useful,” essentially stating that the real test of knowledge is not truth but utility. How can one then make decisions with imperfect models? What steps should we take to make decisions?

The uncertainty associated with model imperfection has implications for how much trust we put in the model’s outcome. Constructing CIs for model uncertainty and observing short lengths of these intervals can provide a measure that facilitates model trust. Short (ie, narrow) CIs necessarily have small magnitude of uncertainty because the length is small. However, it is possible that the magnitude indicated by the length is not acceptable for a given situation, such as, if we need magnitude of <0.5 and we obtain magnitude of 1. Constructing CIs for model uncertainty and observing short lengths of these intervals can provide a measure of understanding the magnitude of the uncertainty and enhancing model trust. Furthermore, computational reproducibility and the existence of protocols that facilitate computational reproducibility adds additional components to model trust. The imperfection of the models used has implications on how to specify, estimate, and evaluate these models as well as for how we interpret the results we obtain and the trust we put in their predictions. Evaluating algorithmic uncertainty via the construction of associated intervals (eg, intervals for assessing predictive uncertainty and model uncertainty) contributes toward better understanding of model performance. The development of a broad collection of models and methods, potentially incorporating varying degrees of uncertainty, provides an approach to decision-making in the presence of imperfect models.

### Discussion and Research Priorities

In this viewpoint, our aim is to describe the lessons learned from the FDA’s experience of applying NLP ML to ICSR assessment and the research needed to address both general issues in case-based reasoning using AI and specific needs for ICSR assessment. Looking through the lens of the Diffusion of Innovations theory, we found that the AI algorithm for deduplication successfully incorporated the key attributes of an innovation, namely, relative advantage, trialability, and observability. The key to the deduplication algorithm’s acceptance is that the tool transparently implemented an algorithm that closely paralleled existing pharmacovigilance workflows and safety reviewers’ cognitive process for deduplication. In contrast, 4 attributes of innovation—compatibility, complexity, observability, and trialability—provide possible explanations for the lack of acceptance of the causality assessment algorithm. These attributes are much more difficult to satisfactorily fulfill for case-based reasoning. From this analysis, we conclude that the lack of a basis for verification of the causality assessment algorithm’s validity in a detailed exposition of a human safety reviewer workflow suggests that developing a more complete understanding of how inferences are made in case-based reasoning for ICSR causality assessment is needed, including improved methods for measuring and controlling of algorithmic uncertainty and computational reproducibility.

It is widely recognized that there remains a gap between AI algorithm development and deployment [1]. Approaches to narrowing the gap are typically presented in the specific technical context of AI algorithms. For example, according to the FDA document on “Artificial Intelligence and Machine Learning (AI/ML) Software as a Medical Device Action Plan,” several factors must be considered when deciding whether an AI algorithm might be ready for implementation. These factors include algorithm performance (eg, validity, generalizability, absence of bias, and robustness in real-world settings with changing inputs), documentation, transparency, explainability (ie, the reasons for an algorithm’s prediction), quality control with real-world data collection and monitoring, and algorithm change control (ie, a structured approach to updating an algorithm using new data) [26]. While AI algorithms used for ICSR processing submission and evaluation by drug companies generally are not required to be submitted to the FDA for approval, the FDA has seen an increase in regulatory submissions with AI components in drug development more generally, including for postmarketing safety monitoring in epidemiological studies [47]. The FDA recently released a discussion paper on AI in drug development to further engage with stakeholders on defining an approach to regulating such algorithms [48].

On the basis of the FDA’s experience of applying AI to ICSR causality assessment, we propose that these factors alone might be insufficient to address the AI development to deployment gap for many case-based reasoning scenarios. Specifically, by applying the Diffusion of Innovations theory, we identify the need for an improved general theory of inference for case-based reasoning as a critical step. In addition, creation of a map of the cognitive framework used by safety reviewers for causality assessment will be necessary for the application of AI to pharmacovigilance.

In moving from these general observations to research priorities to achieve these goals (Textbox 1), we asked ourselves 2 key questions. First, how does the computer “learn” human knowledge? Second, how can we be confident that the knowledge is both correct and accurately captured in the computer algorithms?

Most current approaches answer the first question through a combination of rule-based algorithms, human data annotations, and by using human intervention (ie, the human in the loop) in dialogue with the algorithm to enable iterative learning in machines. Rule-based algorithms are algorithms infused with human knowledge in the form of “if-then” statements for specific rules, and they are not as flexible as ML algorithms. However, rule-based algorithms are used in NLP and are well suited for low data volume and relatively simple rules. A very critical point for developing algorithms of all types is acquiring essential data and annotating them with human intervention. The engagement of the safety reviewer with the computer algorithm can help address the issue of incomplete features and data sparseness, especially when training data are lacking. Thus, the concept “human-in-the-loop” has been proposed to tackle these challenges, by incorporating human knowledge into the process (in this case the algorithmic model).

The answer to the second question is more challenging and currently understudied. New AI technologies using large language models to mimic human reasoning by identifying the most likely sequence of words may become sufficiently robust to superficially meet the Diffusion of Innovation theory’s attributes, such as complexity and compatibility. One such algorithm has already been reported to have passed the medical board examinations and as a result is controversially being proposed as potentially supporting clinical decision-making [50]. In the context of application to case-based reasoning in pharmacovigilance, large language models might be used to summarize the narratives of ICSRs as part of a case series evaluation and to provide a narrative description that has all the hallmarks of a careful analysis but which might be riddled with errors. For example, a large language model might construct a sentence that follows the pattern observed in a narrative describing the relationship between a drug and a clinical outcome. The description, however, might be incorrect because it does not incorporate the scientific and clinical knowledge about the relationships among the various factors that a human expert would likely include in their description. Large language models will likely require that additional knowledge models be incorporated into their workflows for a complete analysis of case series and case-based reasoning more generally.

In conclusion, we need an improved understanding of causal inference and the cognitive framework for determining the causal relationship between a drug and an AE. While human expert evaluation is the current gold standard, the cognitive framework remains incompletely articulated [11,12]. Making a computable cognitive framework trustworthy will not just require its full articulation but also the application of a process to measure and quantify uncertainty as well as computational reproducibility. Implementation should enable transparent comparisons of the data used, the decisions being made by the algorithm to the incorporated data, and decisions made by human experts. Improved understanding of causal inference and the cognitive framework for determining the causal relationship between a drug and an AE will still be important to optimize how such assessments are undertaken and the decision-making process derived from ICSRs regarding the benefits and risks of drugs.

**Research priorities for facilitating the use and trust of artificial intelligence (AI) tools in case-based assessments.**

**For the application of AI to case-based reasoning generally, future work is needed in the following aspects:**

- Evaluate, understand, explain, and ultimately control uncertainty associated with algorithms for them to be useful, using alternative approaches and models.
- Assess computational reproducibility, efficiency, and resource requirements.
- Develop methods that support the evaluation of rule-based algorithms and can measure the extent to which the constructed rules satisfy the end-user’s requirements and that can determine whether the rule definitions are accurate.

**For the application of AI to** **individual case safety report (ICSR) evaluation, future work is needed in the following aspects:**

- Better understand and document the cognitive framework of safety reviewers, in particular, when and how decisions are made using information external to the report itself, such as a case definition or known adverse effects.
- Empirically derive evidence by capturing the actual steps taken by a safety reviewer in real time and developing consensus on the detailed requirements for a high-quality case.
- Develop “tunable” AI algorithms in which performance characteristics and even features such as gender or race used in the algorithm would be selectable by the safety reviewer, consistent with the notion that computational reproducibility, operationalized as “what you can trust and what you can check,” is a key component to the socialization of AI algorithms, which currently has limited formal underlying theory. If the supplied algorithm does not have the flexibility to incorporate these variables and provides results that are different from what the safety reviewer would expect, then the method decreases trust. Qualitative analysis performed by safety reviewers supplies additional variables to incorporate in the revised iteration of the algorithm to improve its performance.
- Explore the relevance of N-of-1 trials in drug development as the closest example of the type of data integration and individualized inferential approach that is needed for case-based reasoning, recognizing that the data limitations of ICSRs make this approach even more challenging. N-of-1 trials are multiple crossover trials, usually randomized and often blinded, and conducted on a single patient. Thus, N-of-1 trials are single-patient trials [49].

## Abbreviations

AE: adverse event
AI: artificial intelligence
FDA: Food and Drug Administration
ICSR: individual case safety report
ML: machine learning
NLP: natural language processing
PML: progressive multifocal leukoencephalopathy
